# Quantitative simulation of near-infrared light treatment for Alzheimer’s disease using patient-individualized optical-parametric phantoms

**DOI:** 10.1117/1.NPh.12.1.015010

**Published:** 2025-02-18

**Authors:** Sihan Dong, Rui Zhang, Jun Xue, Yuanzhen Suo, Xunbin Wei

**Affiliations:** a Peking University, Institute of Medical Technology and Cancer Hospital, Beijing, China; b Peking University, Institute of Advanced Clinical Medicine, Beijing, China; c Peking University, Department of Biomedical Engineering, Beijing, China; d Huashan Hospital, Fudan University, Shanghai Medical College, Department of Neurosurgery, Shanghai, China; e Zhejiang University School of Medicine, Liangzhu Laboratory, Hangzhou, China; f Healthy Life Innovation Medical Technology Co., Ltd, Wuxi, China; g Peking University International Cancer Institute, Beijing, China

**Keywords:** Alzheimer’s disease, near-infrared light treatment, numerical computation, dose–response relationship, neuroimaging analysis

## Abstract

**Significance:**

Alzheimer’s disease (AD) is a brain disorder characterized by its multifactorial nature and complex pathogenesis, highlighting the necessity for multimodal and individualized interventions. Among emerging therapies, near-infrared (NIR) light treatment shows promise as a therapeutic modality for AD. However, existing clinical studies lack sufficient data on light dosimetry, parameter optimization, and dose–response.

**Aim:**

A versatile framework was developed to enable patient-individualized Monte Carlo simulation. A standardized dataset was established, including digital phantoms derived from 20 AD patients who received NIR light treatment.

**Approach:**

The phantoms were synthesized and mapped with multispectral optical parameters, integrating cortical parcellation, subcortical segmentation, and sparse annotation. Structure-related light fluence pathways and dose–response relationships were elucidated using simulation results and cognitive/functional assessments.

**Results:**

The capability for enhancing simulation fidelity and exploring dose–response relationships was verified using standard templates and clinical data. Linear independence was identified between changes in activities of daily living scale scores and energy deposition in gray matter.

**Conclusions:**

The framework offers a solution toward dose–response analysis, parameter optimization, and safety control in the clinical translation for multiple treatment paradigms, demonstrating promise for individualized, standardized, and precise intervention planning.

## 1 Introduction

Alzheimer’s disease (AD) is a progressive neurodegenerative disorder and the most prevalent form of dementia, accounting for an estimated 60% to 80% of dementia cases.^1^^–^^3^ Despite significant advancements in dementia research over the past two decades, 85% of the over 55 million people living with dementia are not receiving adequate post-diagnosis care.^4^^,^^5^ Recent breakthroughs in AD pharmacological therapies, such as the anti-amyloid-beta (anti-Aβ) antibodies lecanemab and donanemab, have shown promising results in reducing Aβ and delaying disease progression.^6^ However, these amyloid immunotherapies are unable to halt the progression of neuronal dysfunction or restore cognitive function.^7^^,^^8^ In addition, the potential side effects, such as amyloid-related imaging abnormalities that can cause seizures and brain bleeding, along with high treatment costs, highlight the urgent need for more effective and safer therapeutic approaches.^7^^,^^9^^,^^10^

Extensive non-pharmacological interventions are emerging as promising combinations or alternative strategies in AD therapy.^11^ With a growing understanding of AD pathogenesis, including immune response, microglia activation, endocytosis, lipid metabolism, and perivascular drainage, non-invasive therapies are gaining attention.^12^^–^^14^ These therapies include photo-biomodulation (PBM), transcranial magnetic stimulation, and transcranial direct current stimulation.^15^^–^^17^ PBM, also known as near-infrared (NIR) light treatment, delivers low levels of red or NIR light with wavelength in the range of 600 to 1000 nm and intensity in the range of 1 to $100\;\mathrm{mW}/{\mathrm{cm}}^{2}$ to AD pathologies or applies pulsed visual stimulations.^18^^,^^19^ PBM has been shown to enhance mitochondrial function, reduce neuroinflammation, promote adult hippocampal neurogenesis, and improve glymphatic drainage.^20^^–^^22^ As a non-invasive, low-cost, and home-care suitable therapy, NIR light treatment shows potential in preventing and alleviating AD-associated pathology and cognitive decline without significant side effects.^23^^,^^24^

Accumulating evidence from preclinical studies and clinical trials indicates that different NIR light treatment paradigms significantly influence therapeutic outcomes.^20^^,^^25^^,^^26^ Therefore, optimizing treatment parameters, personalizing therapeutic schedules, and precisely evaluating dose–response relationships are crucial for successful treatment. However, clinical application faces challenges in quantitatively and non-invasively measuring light penetration and energy distribution in various brain regions.^27^^,^^28^ Therefore, it is necessary to develop accurate, efficient, and standard strategies to evaluate light transmittance, optimize illumination parameters, and maximize therapeutic efficiency. With advancements in numerical computation technology, algorithms based on Monte Carlo simulation (e.g., MCML, tMCimg, MCX) have provided solutions to these challenges.^29^^–^^31^ The optical Monte Carlo method is widely considered the gold standard for studying light propagation in biological tissues as it enables accurate quantitative calculation of the dose delivered to deep tissues and provides guidance for clinical trial design, offering advantages such as flexibility, efficiency, and low cost.^32^^–^^34^ However, its limitations, including (1) the lack of AD-specific phantoms for dose–response analysis, (2) discretization or staircase artifacts at tissue boundaries that compromise simulation fidelity, and (3) the luck of standardized simulation strategies for longitudinal or interindividual analyses, significantly affect calculation accuracy and hinder clinical translation.^35^^,^^36^

This study aims to construct a versatile Monte Carlo simulation framework and validate it using optically heterogeneous phantoms derived from AD patients recruited in a pilot clinical trial.^24^ The framework offers an unprecedented capability to simulate sophisticated treatment paradigms and assesses their safety, such as light propagation during trans-ocular interventions, by mapping multi-wavelength optical parameters to brain and extra-cerebral tissue within the phantoms aligned to a standard coordinate space, while accounting for physiological differences among patients. By integrating advanced neuroimaging analysis and registration algorithms, the framework efficiently identifies light fluence pathways through brain regions of interest (ROIs) and explores their structure-specific energy deposition patterns and dose–response relationships, all while minimizing manual labeling workload. This strategy aims to pave the way for precise NIR light treatment, automated planning, safety control, and dose–response analysis in clinical applications.

## 2 Materials and Methods

### 2.1 Numerical Phantom

**Fig. 1:**
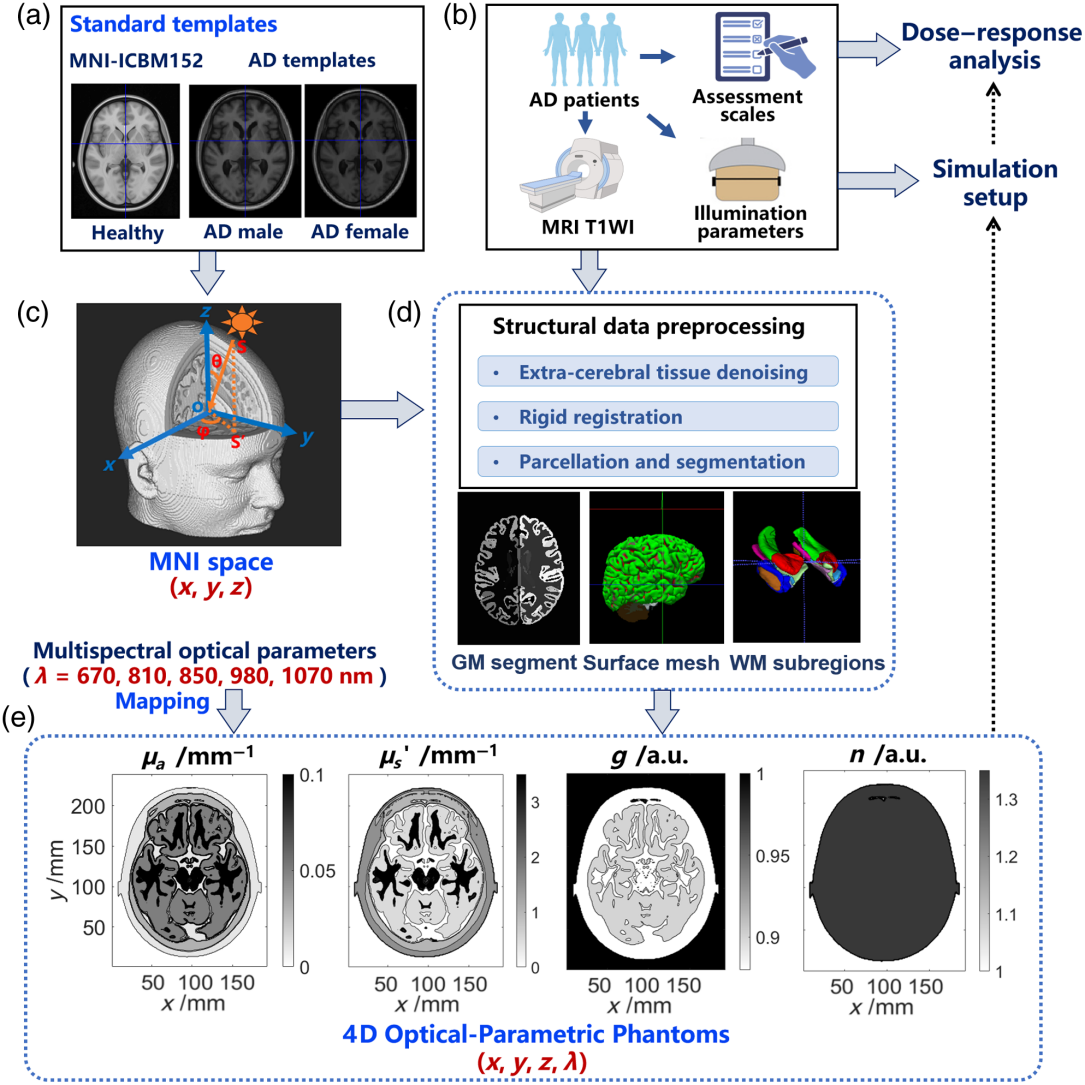
Schematic diagram of the framework for synthesizing optical-parametric numerical phantoms. (a) T1WI data of standard templates, including MNI-ICBM152 and AD-specific male/female templates. (b) Patient data collection process for subsequent simulation and dose–response analysis, including cognitive metrics, demographic information, treatment parameters, and T1WI data of 20 AD patients. (c) The MNI standard space and Cartesian coordinate used for spatial normalization of phantoms and location of light sources. The direction of light sources can be determined by angles φ (azimuth angle) and θ (polar angle), or manually set to ensure the tangibility to skin surfaces. (d) Preprocessing of patient phantoms using neuroimaging tools, including tissue denoising, gray matter (GM) parcellation, anatomical feature extraction, and segmentation of white matter (WM) subregions, such as the medial temporal lobe. (e) Multispectral optical parameters mapping onto the numerical phantoms. Wavelength (λ): 670, 810, 850, 980, 1070 nm; optical parameters: absorption coefficient ($\mu_{a}$), reduced scattering coefficient ($\mu_{s}^{\prime }$), anisotropy factor (g), and refractive indices (n); Contour map: horizontal cross-section (vertical coordinate, z=0, λ=810  nm) of the standard average phantom synthesized using MNI-ICBM152 templates.

The framework for synthesizing optical-parametric numerical phantoms was developed and detailed in Fig. 1. Initially, we leveraged raw T1-weighted imaging (T1WI) data from MNI-ICBM 152 templates to synthesize a standard extended phantom [Fig. 1(a)].^37^^,^^38^ This phantom incorporates extended anatomical regions, including the nasal cavity, oral cavity, and orbital cavity, facilitating the reconstruction of extracranial tissues and enabling the simulation of trans-nasal and trans-oral illumination paradigms. To further enhance disease-specific representation, standard AD male and female phantoms were synthesized from multi-sequence average templates in the COMPASS-ND dataset, ensuring an accurate representation of each sex group.^39^ In addition, the standard templates were used for spatial normalization and registration of patient-specific phantoms. Given that T1WI data are typically acquired in individual (subject-specific) space with various sizes, resolutions, and coordinate directions when collected in different clinical settings [Fig. 1(b)], the standard templates with Montreal Neurological Institute (MNI) space were set as the target for T1WI registration.^40^ As shown in Fig. 1(c), the coordinate origin in the space is chosen at the anterior commissure, with the negative Y-axis passing through the posterior commissure. The Cartesian coordinate system was integrated into the space to ensure comparability of brain anatomy and accuracy of light source positioning across different AD patients.

The preprocessing of the T1WI structural phantoms involves three main steps [Fig. 1(d)]. First, we removed external metal artifacts and noise from the extra-cerebral tissues, including the skull and scalp. We located the extra-cerebral tissues and identified artifacts isolated out of the tissue boundaries based on the volume and connectedness of the artifact components using the Image Processing Toolbox in MATLAB 2023a. Then, we further refined the removal of attached artifacts by applying manual calibration based on the tissues’ anatomical continuity and connectivity. Second, we employed a robust registration method to rigidly align the phantoms to the MNI standard space, ensuring the anatomical integrity and consistency of brain ROIs without introducing distortions.^41^ This registration method was also applied to align phantoms derived from different visits of AD patients during our clinical trial, enabling motion correction and longitudinal analysis of the same subject. Third, we performed cortical segmentation, sub-cortical parcellation, and anatomical feature extraction across multiple brain ROIs using neuroimaging software, including FreeSurfer (version 7.2.0),^42^^,^^43^ SPM (version 12),^44^ and ITK-SNAP (version 4.2.0).^45^ By integrating these tools, we improved the tracking of brain volume changes, clarified light fluence pathways, and quantified energy deposition within specific regions.

After the preprocessing, the structural phantoms are composed of voxels indexed by three-dimensional matrices with a spatial resolution of $1\times 1\times 1\;mm^{3}$, typically identical to the source magnetic resonance imaging (MRI) data. Moreover, the likelihood that a given voxel belongs to a particular tissue type, including gray matter, white matter, cerebrospinal fluid, skull, soft tissues (including dura mater, scalp, muscle, etc.), and air within the sinuses, was determined by the probabilistic models from the SPM software within MNI space. As shown in Fig. 1(e), optical parameters, including the absorption coefficient ($\mu_{a}$), scattering coefficient ($\mu_{s}$), anisotropy factor (g), and refractive indices (n), were mapped to each voxel within the phantoms. These optical parameters are wavelength-dependent and vary significantly among different tissues. We calculated a weighted average of the optical parameters within each voxel for heterogeneous tissues by adopting tissue-specific optical parameters reported in the previous studies, including skin and subcutaneous/mucous tissues,^46^^,^^47^ cranial bone,^48^^,^^49^ brain white and gray matter,^50^ cerebrospinal fluid and fat tissue,^34^^,^^51^ and visual organs.^52^^,^^53^ The weights were derived from probabilistic maps of tissue types segmented using the neuroimaging software or expert annotations, thus enhancing fidelity and continuity at tissue boundaries within the synthesized phantoms. To ensure precise alignment and calibration of optical parameters in the heterogeneous medium involving the tissues of the eyes and orbitals, sparse annotations were conducted. Specifically, based on the signal intensity and structural characteristics of T1WIs, an experienced radiologist performed a detailed identification of fat, muscle cortical bone, and vitreous body to manually sample and segment voxels of different tissue types using the NII-Viewer toolbox in MATLAB 2023a. Subsequently, we further improved the continuity and smoothness of the structural details at the tissue boundaries using conventional linear interpolation and Hermite interpolation in the orbital tissues, as described in Ref. 54. The phantoms are termed four-dimensional because they not only possess spatial anatomical features but also include spectral features of the optical parameters. In this work, we synthesized multispectral phantoms with wavelengths of 670, 810, 850, 980, and 1070 nm, which are typically used in NIR light treatment for AD patients.

### 2.2 MC Simulation Setup

We implemented a quantitative Monte Carlo method to model light propagation through the optically heterogeneous phantoms to target brain regions based on the open-source simulation software MCX.^31^ The main simulation parameters are listed in Table 1. Initially, we performed on the extended phantom to explore illumination outcomes of various treatment paradigms. For non-invasive paradigms, including transcranial and trans-ocular illuminations, the source radius was set to 10 mm, whereas for minimally invasive paradigms, including trans-nasal and trans-oral, that was 5 mm to enhance anatomical conformity. The total absorbed energy was normalized before analyzing the simulation outcomes of these paradigms. Subsequently, we performed simulations using 10 uniform planar light-emitting diodes (LEDs) simultaneously illuminating the AD phantoms, with the source wavelengths, positions, sizes, and power density identical to those applied in the clinical treatment. As shown in [Figs. S1(g) and S1(h) in the Supplementary Material], for the multi-source treatments conducted on AD patients, the illumination positions targeted brain regions corresponding to the 10 to 20 international system.^55^^,^^56^ The positions were set as follows: R frontal was located at F4–Fz–Fp2, whereas L frontal was at F3–Fz–Fp1. R ocular was positioned at Fp2–Fz and right eye, whereas L ocular was at Fp1–Fz and left eye. R tempus was designated at F8, and L tempus was at F7. R parietal was placed at P3–Pz, and L parietal was at P4–Pz. R occipital was located at O1, and L occipital was at O2. The skins in these illumination positions have flat surfaces, making it compatible with the LED light arrays. In addition, hair and hair follicles in these positions have minimal absorption of near-infrared light, resulting in less photon energy loss. To minimize simulation error, the number of photon packets was set to $5\times 10^{7}$ to balance accuracy, reliability, and time cost, based on an empirical formula derived from Ref. 57. This number reflects the total illumination energy of the light source and can achieve stable light fluence output for the same simulation setup. The simulation runs on the Nvidia RTX 4090 GPU leveraging CUDA acceleration for efficient computation, with an average time cost of ∼5  min per patient. Furthermore, the temporal resolution to record the trajectory of photons was set to 0.3 ns for time-resolved simulation, and the photons are considered waves at each scattering location while being treated as classical particles elsewhere. The coherence, polarization, and nonlinearity of the photons are ignored. The structural anisotropy of tissue components is also disregarded to focus on the deposited energy within the tissue.^29^

**Table 1:** Simulation parameters for modeling light propagation in the phantoms (R, right; L, left).

Parameters	Extended phantom	AD phantom
Wavelength	670, 810, 850, 980, 1070 nm	810, 1070 nm
Illumination position	Forehead, head top, occipital, eyes, oral cavity, and nasal cavity see [Figs. S1(a)–S1(f) in the Supplementary Material]	R/L frontal, R/L ocular, R/L tempus, R/L parietal, and R/L occipital see [Fig. S1(g) in the Supplementary Material]
Source type	Disk-shaped LED (radius: 10 mm for non-invasive sources and 5 mm for minimally invasive sources)	Rectangular LED arrays (size: 40 mm × 34 mm)
Power density	$20\;\mathrm{mW}/{\mathrm{cm}}^{2}$	$20\;\mathrm{mW}/{\mathrm{cm}}^{2}$
Number of photon packets	$5\times 10^{7}$	$5\times 10^{7}$

### 2.3 Participants and Data Collection

We collected cognitive and functional scales and T1WI data from 20 participants with mild to moderate AD recruited in our previous clinical study^24^ [Fig. 1(b)]. Written informed consent was obtained from all the participants or their guardians. The Alzheimer’s disease assessment scale-cognitive (ADAS-Cog), the activities of daily living (ADL), and the Mini-Mental State Examination (MMSE) were assessed at baseline, weeks 4, 8, and 12 of the treatment. Twenty participants with mild to moderate AD were randomly assigned to the intervention or control group in a 1:1 ratio. The baseline demographic and clinical characteristics of the intervention group (10 participants; age (y), 68.9±9.05; race Asian, 10; men, 4; women, 6; ADAS-Cog, 20.0±8.98; ADL, 19.0±5.14; MMSE, 20.2±4.98) showed no significant differences compared with the control group (10 participants; age (y), 72.1±8.46; race Asian, 10; men, 5; women, 5; ADAS-Cog, 18.4±5.76; ADL, 21.2±6.80; MMSE, 18.5±3.21). The intervention group underwent NIR light treatment with wavelengths of 1060 to 1080 nm and 800 to 820 nm for 12 weeks. The T1WI data of the AD patients were collected using the same system that operates at 3 Tesla using a 3D gradient echo and inversion recovery sequence, with a repetition time of 2530 ms, an echo time of 2.98 ms, a flip angle of 7 deg, and an inversion recovery sequence order, providing high-resolution imaging with enhanced contrast. More detailed information, such as the scale scores at each visit, can be found in our previously published study^24^ or obtained upon request from the corresponding author. The approval information from the Medical Ethics Committee of Huashan Hospital is provided in the Acknowledgments section.

### 2.4 Statistical Analysis

To investigate the responses of different brain regions to light stimulation, we analyzed the energy deposition and distribution patterns across over one hundred brain regions using 10 light sources. The brain region annotation and structural feature extraction were performed using the FreeSurfer software according to Desikan–Killiany atlas.^58^ Sankey diagrams were used to visualize light propagation pathways within the AD average and patient-specific phantoms, effectively representing the energy distribution, as well as the relative intensity and direction of light propagation. A correlation matrix was computed using the Pearson correlation coefficients to examine energy distributions. To identify intrinsic grouping patterns, hierarchical clustering analysis was conducted at various levels of granularity for parcellated regions using MATLAB’s Statistics and Machine Learning toolbox. This analysis evaluated similarity based on the unweighted average Euclidean distance between brain region pairs, with results visualized through dendrograms. This clustering approach, which requires no predefined number of clusters, is robust for handling clusters of varied sizes and is suitable for exploratory analyses, such as functional connectivity analysis of brain regions.^59^ When accessing therapeutic effects using ADAS-Cog, ADL, and MMSE and comparing light energy deposition in different regions or tissues for different sex groups and light wavelengths, analysis of variance was used to compare these continuous variables. If the variables followed normal distributions (Kolmogorov–Smirnov test: p>0.05) and have equal variances (Levene’s test: p>0.05), independent samples t-tests were performed for sex group comparisons, and two-tailed paired t-tests for intra-individual phantom comparisons. Otherwise, the Wilcoxon rank-sum test was employed, which is better suited for small sample sizes. p values and 95% confidence intervals were estimated in two-tailed tests, with statistical significance set at p<0.05. The mean values and standard deviations were used for the statistical description of baseline demographic and clinical characteristics of the intervention and control groups. Given the exploratory nature of this pilot study, some participants either did not complete or prematurely ended follow-up visits, resulting in missing data for cognitive and functional assessments. To maintain statistical power, dose–response relationships were primarily analyzed using ADAS-Cog, ADL, and MMSE scores at baseline and week 8 via linear regression analysis. Regression and correlation coefficients were tested using two-tailed t-tests. To evaluate therapeutic effects on AD-related regional volumes and to examine correlations between scale scores and brain structural or dosimetric features, we analyzed structural changes and dosimetric differences in eight AD patients with comprehensive T1WI data and intervention scales at baseline and week 12. All statistical analyses and visualizations were performed using MATLAB 2023a (The MathWorks, Natick, Massachusetts, United States).

### 2.5 Quality Control

**Fig. 2:**
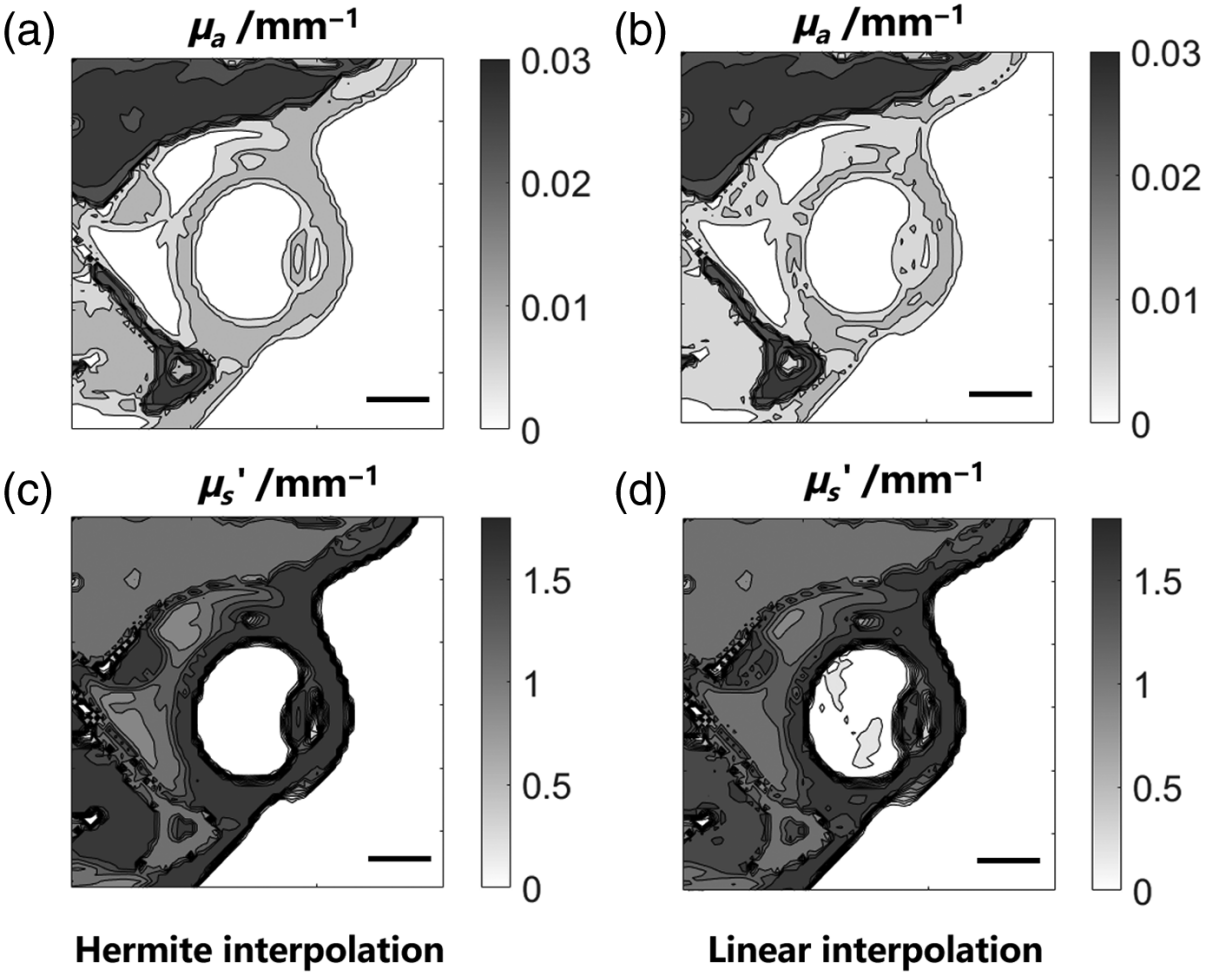
Absorption coefficient ($\mu_{a}$) and reduced scattering coefficient ($\mu_{s}^{\prime }$) mapped onto the visual organs within the numerical phantoms. The $\mu_{a}$ was mapped by combining manual labeling with (a) Hermite interpolation methods or combining the same labeling with (b) linear interpolation. The $\mu_{s}^{\prime }$ was mapped by combining manual labeling with (c) Hermite interpolation methods or combining the same labeling with (d) linear interpolation. (a)–(d) Scale bar, 10 mm; contour map: horizontal cross-section through the geometric center of the right eye; unit of the $\mu_{a}$ and $\mu_{s}^{\prime }$ contour map: $1\;{\mathrm{mm}}^{-1}$; wavelength: 810 nm.

**Fig. 3:**
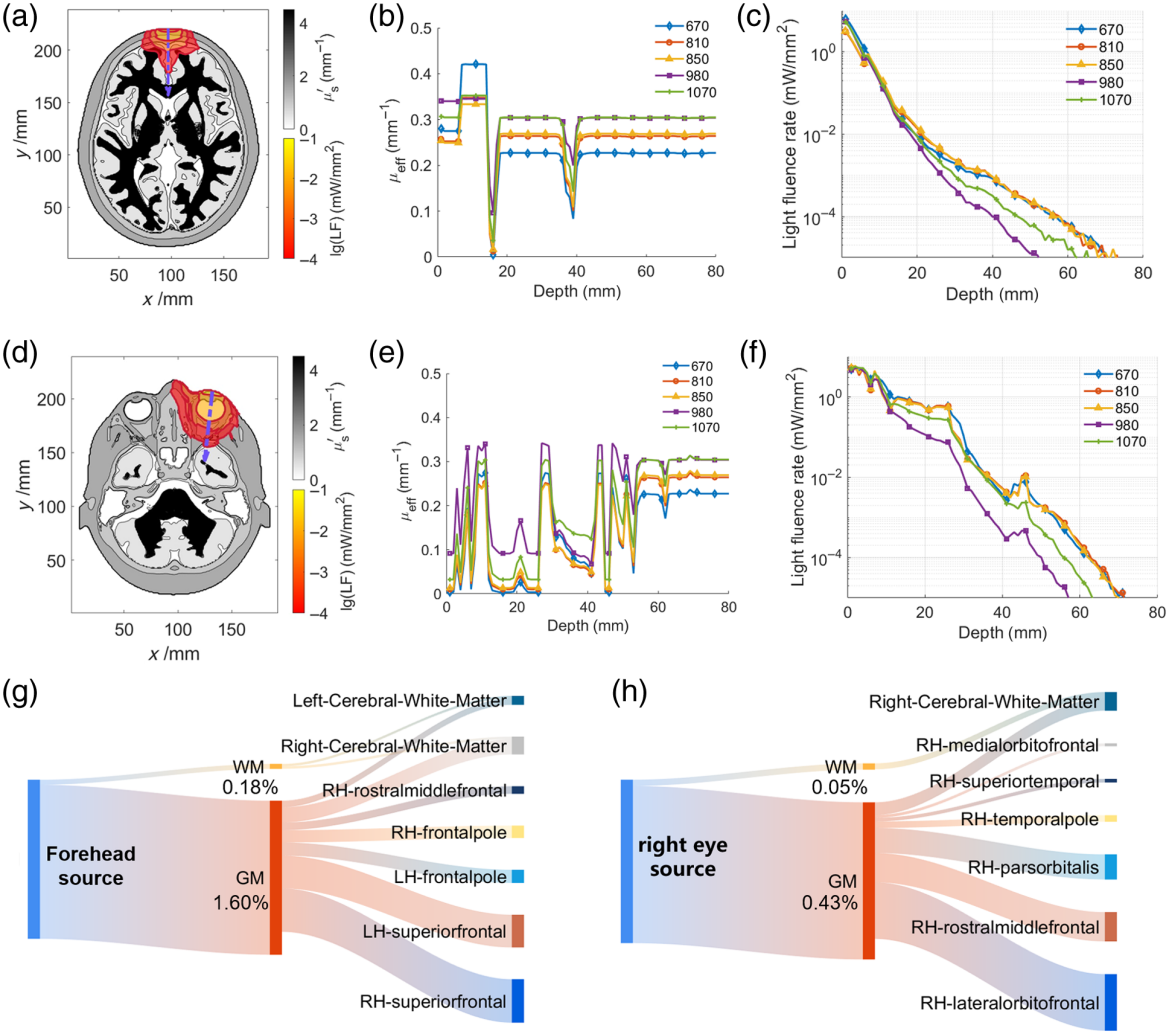
Light fluence rate distribution and light propagation pathways revealed by multi-wavelength simulation using the extended optical-parametric phantom. (a) Horizontal plane of light fluence (LF) rate distribution and reduced scattering coefficient ($\mu_{s}^{\prime }$) map in the numerical phantom during transcranial intervention from the forehead. The violet dotted arrow indicates the projection of the illumination direction on the horizontal cross-section. (b) Effective attenuation coefficient ($\mu_{\mathrm{eff}}$) along the transcranial illumination direction in the phantom. (c) Light fluence rate along the transcranial illumination direction in logarithmic (lg) scale. (d) Horizontal plane of light fluence rate distribution and $\mu_{s}^{\prime }$ map in the numerical phantom during trans-ocular intervention from the right eye. Violet dotted arrow indicates the projection of the illumination direction on the horizontal cross-section. (e) $\mu_{\mathrm{eff}}$ along the trans-ocular illumination direction in the phantom. (f) Light fluence rate along the trans-ocular illumination direction in logarithmic (lg) scale. (g), (h) Sanky diagram illustrating photonic energy flow and deposition pathways within brain white matter (WM), gray matter (GM), and cortical regions during (g) transcranial intervention from the forehead and (h) trans-ocular intervention from the right eye (Video 1, MP4, 8.65 MB [URL: https://doi.org/10.1117/1.NPh.12.1.015010.s1]).

The quality of the registrations, the pre-processed images, and the volumetric segmentations performed by the neuroimaging techniques were assessed visually by an experienced rater (MD). All images passed this quality control step. In addition, the source MRI data were quality controlled by professional imaging physicians at Shanghai Huashan Hospital for the presence of imaging artifacts, and only scans that passed this quality control step were acquired and used for this study. In terms of qualitative comparison with other atlases in the field, based on visual assessment, the provided phantoms have a high level of image sharpness and anatomical detail, clearly delineating the sulci and gyri in the cortex [Figs. 1(e), 2, 3(a), 3(d), 5(a), and 5(b)].

## 3 Results

### 3.1 Simulation Framework Enhances the Reconstruction Fidelity and Facilitates Safety Assessment

Initially, we validated the simulation capabilities of the established framework for novel treatment paradigms using the extended phantoms derived from the MNI-ICBM 152 atlases, including trans-ocular NIR light treatments administered via two LED arrays, referred to as the R ocular and L ocular. In the clinical study, the two source arrays were positioned in front of each patient’s eyes to stimulate deeper brain regions, such as the orbitofrontal cortex, entorhinal cortex, and hippocampus. Thus, a precise calculation of energy deposition in the target regions, as well as ensuring the treatment’s safety for the visual organs, requires accurate mapping of optical parameters within the visual organs. As shown in Fig. 2, we developed a method combining manual sparse annotation with Hermite interpolation to label the optical parameters accurately and efficiently. The sparse annotation involved manually identifying a limited number of voxels or areas in T1WIs, including the eyeballs, eyelids, and orbital fat. This method leverages prior knowledge that the optical parameters satisfy natural boundary conditions, ensuring continuity of the gradients at tissue boundaries.^60^ Compared with linear interpolation, Hermite interpolation enables the creation of more accurate and realistic phantom structures of the corneas, lens, vitreous body, and ciliary ligament. The lamellar structure of the lens and the lens nucleus are clearly visible in Figs. 2(a) and 2(c), whereas they are difficult to discern in Figs. 2(b) and 2(d). This approach facilitates a more precise depiction of ocular structures, suppresses staircase artifacts and discrete error, and preserves enhanced optical-parametric continuity and smoothness at the tissue boundaries.

Subsequently, we conducted simulations of light propagation through visual organs and extracerebral tissues (Fig. S2 in the Supplementary Material and Fig. 3) and thus performed a quantitative safety analysis. Although the safety of low-level red or NIR light treatment for myopia has been reported, research on its safety in the treatment of AD remains limited.^61^^,^^62^ In the intervention group of our previous clinical study, one device-related adverse event (mild conjunctivitis) was reported in one AD patient, accounting for an adverse event rate of 10% among the 10 participants. This patient had multiple concomitant diseases, including diabetes, a known risk factor for developing conjunctivitis.^63^ Although the adverse event was mild and resolved without sequelae after medication and was more likely attributable to the underlying diseases, it underscores the importance of quantitatively assessing trans-ocular treatment paradigms. Therefore, we assessed the safety of NIR light for the skin, cornea, and retina based on the simulation results and the American National Standard for Safe Use of Lasers (ANSI Z136.1).^64^ According to the standard, the maximum permissible exposure (MPE) criteria for skin exposure is $10.00\;\mathrm{mW}/{\mathrm{mm}}^{2}$ for 1070 nm and $3.32\;\mathrm{mW}/{\mathrm{mm}}^{2}$ for 810 nm, which are at least 10 times higher than the power density of the LED arrays ($0.20\;\mathrm{mW}/{\mathrm{cm}}^{2}$), given that the LED light sources for trans-ocular therapy can be considered extended sources (with an angular subtense, α, greater than 100 mrad), due to the beam divergence and light diffusion within the skin.^65^ As shown in Fig. S2 in the Supplementary Material, the light fluence rate reaching the front of the cornea is $0.136\;\mathrm{mW}/{\mathrm{mm}}^{2}$ for 1070 nm and $0.112\;\mathrm{mW}/{\mathrm{mm}}^{2}$ for 810 nm, both of which are below the ANSI standard for extended-source ocular exposure MPE (i.e., less than $1.90\;\mathrm{mW}/mm^{2}$ for 1070 nm and $0.63\;\mathrm{mW}/{\mathrm{mm}}^{2}$ for 810 nm). Furthermore, we analyzed the light fluence rate at the retina, which is $1.60\times 10^{2}\;\mathrm{mW}/{\mathrm{mm}}^{2}$ for 1070 nm and $3.04\times 10^{2}\;\mathrm{mW}/{\mathrm{mm}}^{2}$ for 810 nm, which is two orders of magnitude lower than the safe power density of $5\;\mathrm{mW}/{\mathrm{mm}}^{2}$ reported by Lorach et al.^66^ This power density was found to induce thermal effects with temperature increases ranging from 0.17°C to 0.43°C on the retina, well below the recommended thermal safety limit of 2°C according to the international standard (ISO 14708-1).^67^

### 3.2 Simulation Framework Reveals Light Pathways Within Brain ROIs During Multiple Treatment Paradigms

We further validated the efficacy and transferability of the framework across multiple treatment paradigms using extended phantoms, prior to applying it to patient phantoms derived from clinical data. The treatment paradigms involved light propagation through various anatomical structures, including nasopharyngeal regions, oral cavities, and ocular orbits. The light fluence rate and distribution of optical parameters along illumination directions, as well as the pathways of light propagation within cortical ribbons and sub-cortical structures during transcranial and trans-ocular paradigms, are visualized in Fig. 3.

For the transcranial treatment paradigm applied via the forehead, when the light fluence rate decreases by three to four orders of magnitude from its initial value, it penetrates the gray matter and white matter, respectively [Fig. 3(a), Video 1]. The contour map of the reduced scattering coefficient ($\mu_{s}^{\prime }=\;(1-g)\mu_{s}$) highlights the anatomical structure and weak absorption features of the brain’s longitudinal fissure, facilitating deep light propagation along the path. Moreover, the effective attenuation coefficient ($\mu_{\mathrm{eff}}$) along the light path was calculated using $\mu_{\mathrm{eff}}=\sqrt{3\mu_{a}(\mu_{a}+\mu_{s}^{\prime })}$, accounting for the combined effects of absorption and scattering in highly scattering media.^68^ As illustrated in Figs. 3(b) and 3(c), at depths ranging from 0 to 14 mm, light passes sequentially through the scalp and skull, with a light fluence rate attenuation of two orders of magnitude. At depths of 14 to 40 mm, the cerebrospinal fluid within the longitudinal fissure mitigates light attenuation across five wavelengths, enhancing the penetration depth to 40 to 56 mm as the light fluence rates diminish to $10^{-4}\;\mathrm{mW}/{\mathrm{mm}}^{2}$.

Similarly, the trans-ocular paradigm was simulated to provide insights into light propagation from the right eye into deeper brain regions, such as the temporal lobe, as shown in [Fig. 3(d)]. The $\mu_{\mathrm{eff}}$ extracted along the trans-ocular path clearly demonstrates the attenuation effects on light propagation within different layers of tissue [Figs. 3(e) and 3(f)]. At depths ranging from 0 to 11 mm, light at all five wavelengths is significantly attenuated by the eyelids, iris, and ciliary body, whereas only slightly attenuated by the cornea and eyeball chambers, causing the $\mu_{\mathrm{eff}}$ curve to exhibit oscillatory behavior. From 12 to 41 mm, the vitreous body and orbital fat create two optical windows that facilitate light propagation to deeper tissues, whereas the retina, sclera, and fascial sheath of the eyeball form a $\mu_{\mathrm{eff}}$ peak between the two windows. Finally, beyond 42 mm, light propagates through the accessory organs of the eye, skull, and cerebrospinal fluid and ultimately reaches the brain’s gray matter.

Subsequently, we utilized Sankey diagrams to compare photon transport pathways and energy deposition during the two paradigms [Figs. 3(g) and 3(h)]. For the forehead source, 1.78% of the total energy was absorbed by the brain parenchyma, primarily in regions such as the white matter, superior frontal areas, and frontal poles of both hemispheres. By contrast, the right-eye source resulted in 0.48% of the total energy being absorbed by the brain parenchyma, mainly deposited in the right cerebral white matter, lateral orbitofrontal regions, rostral middle frontal, and pars orbitalis. These results demonstrate that source positions significantly influence energy distribution, potentially targeting different symptoms and offering distinct benefits for treating AD.

In addition, we investigated the potential for targeted intervention in deep brain regions, including the amygdala and hippocampus, both located in the medial temporal lobe (MTL) region, by quantifying the average light fluence rate within these regions during various NIR light treatment paradigms (Fig. S3 in the Supplementary Material). The temporal point spread function (tPSF) was calculated using time-resolved Monte Carlo simulations. The multi-wavelength tPSFs presented in [Figs. S3(a) and S3(b) in the Supplementary Material] illustrate the temporal distribution of a unit light pulse propagating in the amygdala region. At the tPSF peak, transcranial paradigms with wavelengths of 850 and 810 nm achieved higher amplitudes within ∼0.6  ns, whereas trans-ocular paradigms with wavelengths of 810 and 670 nm achieved higher amplitudes within ∼1.5  ns. These differences are attributed to variations in light pathways caused by the distinct structures and optical properties of brain and extracerebral tissues [Figs. S3(c)–S3(f) in the Supplementary Material]. Besides, the average light fluence rates under different illumination directions indicate that the wavelengths of 670 and 810 nm provide superior stimulation intensity in the bilateral hippocampus regions [Fig. S3(g) in the Supplementary Material]. These findings suggest that optimizing illumination parameters, such as light intensity, pulse trigger timing, and duration, holds promise for precisely targeting deep brain regions, including MTL, thereby enhancing the therapeutic efficacy of NIR light treatment.

Furthermore, we compared the energy deposition for the six typical treatment paradigms illustrated in [Figs. S1(a)–S1(f) in the Supplementary Material]. According to the results in Table 2, the transcranial (forehead) paradigm results in the highest gray matter energy deposition for 810 nm (1.60%), and the trans-ocular and transcranial (head top) paradigms also show relatively higher energy deposition in gray matter (0.43% and 0.74%, respectively), whereas the trans-oral and trans-nasal paradigms have minimal energy deposition in gray matter. The transcranial (head top) paradigm is more effective for energy deposition in white matter (0.33%) compared with other paradigms. Besides, the forehead paradigm also shows moderate white matter energy deposition (0.18%), making it a viable option for white matter stimulation.

**Table 2:** Energy deposition during various treatment paradigms with a wavelength of 810 nm.

Treatment paradigm	Transcranial (forehead) (%)	Trans-ocular (%)	Transcranial (head top) (%)	Transcranial (occipital) (%)	Trans-oral (%)	Trans-nasal (%)
Gray matter	1.601	0.433	0.736	0.063	0.001	0.030
White matter	0.183	0.053	0.330	0.002	0.001	0.004
Cerebrospinal fluid	0.282	0.058	0.278	0.006	0.001	0.009
Skull	26.220	11.697	11.734	0.866	44.239	73.110
Soft tissues (scalp, muscle, etc.)	71.715	87.759	86.922	99.063	55.758	26.848

### 3.3 Simulation of Multi-Source Interventions on AD Phantoms Reveals Clustering Characteristics and Sexual Difference of the Dose Distribution

**Fig. 4:**
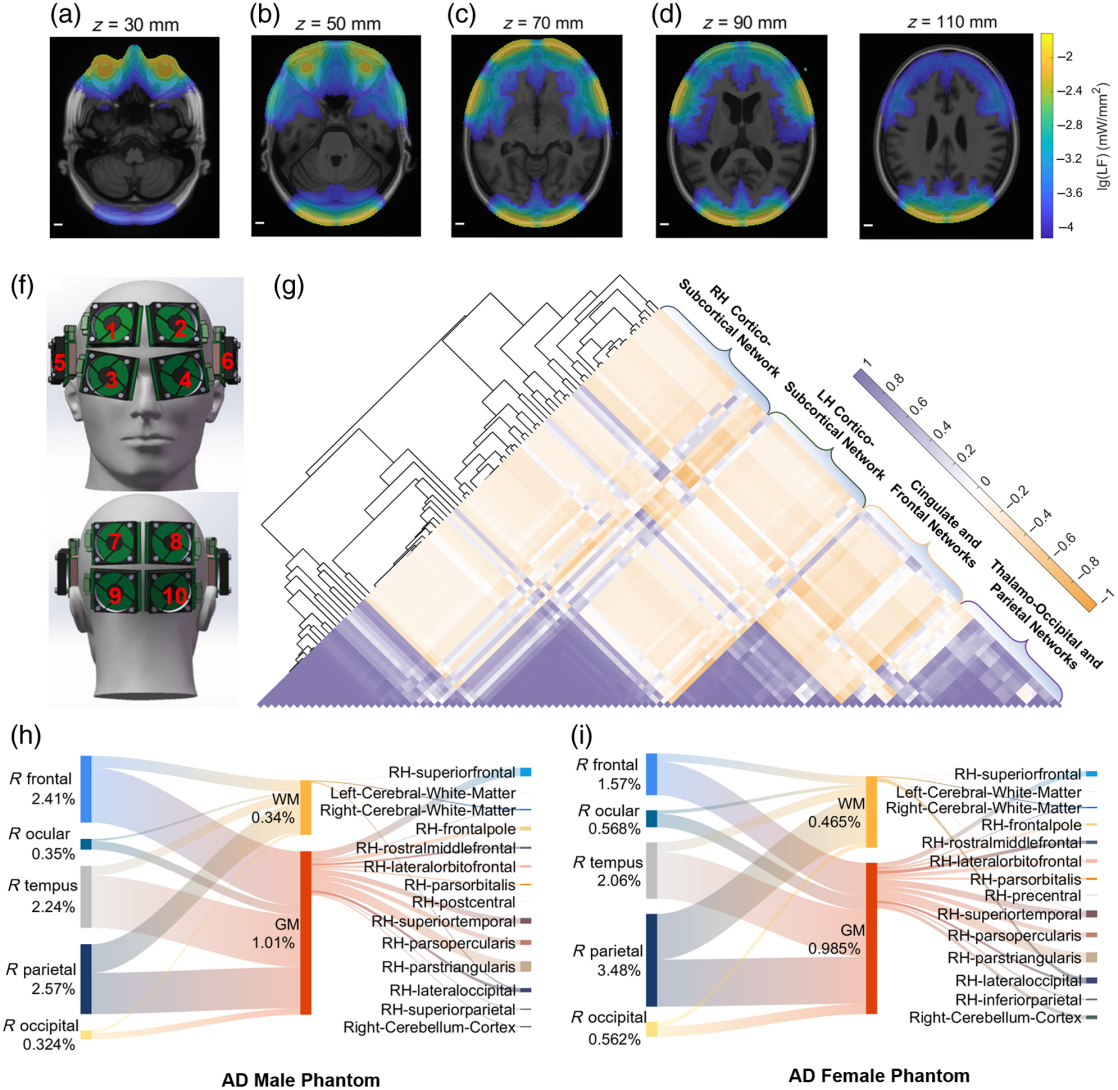
Multi-source simulation based on the AD-specific phantom derived from the average neurodegenerative brain templates. (a)–(e) Light fluence rate distribution mapped to horizontal planes of T1WIs (z=30, 50, 70, 90, 110 mm) in MNI standard space; light wavelength, 810 nm; scale bar, 10 mm. (f) Schematic diagram of the ten LED arrays used for transcranial and trans-ocular intervention. Numbers, 1/2: R/L frontal; 3/4: R/L ocular; 5/6: R/L tempus; 7/8: R/L parietal; and 9/10: R/L occipital. (g) The heatmap of pairwise Pearson correlation coefficient for responses of different brain regions to light stimulation. Dendrogram: grouping clusters of brain regions that exhibit similar energy accumulation patterns. Brain ROIs: 98 regions annotated using Desikan–Killiany, which were divided into four main clusters using hierarchical clustering (see Fig. S4 in the Supplementary Material). (h), (i) Sanky diagrams illustrating sexual difference of the dose distribution and energy flow pathways within WM and GM from five light sources targeting the right hemisphere between (h) average male and (i) average female AD phantoms (Video 2, MP4, 642 KB [URL: https://doi.org/10.1117/1.NPh.12.1.015010.s2]).

The above-mentioned extended phantoms are based on a cohort of healthy subjects and cannot represent the anatomical characteristics of AD populations. To realize precise dose evaluations and individualized intervention planning for AD patients, we synthesized AD phantoms using the average templates of neurodegenerative male and female patients for pre-simulation.^39^ We implement the multi-source simulation with ten LED light sources consistent with the location, orientation, size, and power density of the LED array embedded in the therapeutic device used in the clinical pilot study [Table 1, Fig. S1(g) in the Supplementary Material].^24^ First, we leveraged average AD phantoms derived from templates of all sex groups to assess the light dose distribution within AD-specific brain structures. As illustrated in Figs. 4(a)–4(e), the light fluence maps at z=30, 50, 70, 90, and 110 mm, merged with horizontal cross-sections of T1WIs, demonstrate the synergistic modulation effect of the 10 light sources. This configuration enables light to penetrate into brain regions deeper than those achieved by the single-source paradigms described earlier. These findings inspired further investigation into the dose distribution across structurally and functionally related brain regions and their responses to stimulation from the 10 LED light sources [Fig. 4(f)].

To explore the intrinsic grouping patterns of the brain regions segmented and parcellated according to the Desikan–Killiany brain atlas, we utilized hierarchical clustering analysis to divide the regions into four main groups, each characterized by similar responses to light stimulation (i.e., the average light fluence rate induced by each light source) [Fig. 4(g)]. For example, the group named the right hemisphere cortico-subcortical network containing the right-hemisphere temporal lobe, right hippocampus, right amygdala, and entorhinal cortex, which are structurally and functionally interconnected regions involved in memory processing, emotional regulation, and spatial navigation [Fig. S4 in the Supplementary Material].^69^ The Pearson correlation coefficients of the average dose distribution between these regions were generally greater than 0.80, indicating a strong positive relationship. This suggests that these regions exhibit similar energy accumulation patterns, potentially reflecting a shared intervention pathway and dose–response mechanism during NIR light treatment in AD patients. Conversely, the light fluence rates in regions within the aforementioned group generally show a negative correlation with those in the group named cingulate and frontal networks, likely due to their greater anatomical separation. This finding indicates that these regions may require distinct illumination parameters to achieve optimal therapeutic effects.

In addition, we examined sex differences in dose distribution using Sankey diagrams [Figs. 4(h) and 4(i)]. Given the symmetry of the average brain phantoms, the diagrams illustrate the dosimetric contributions of five NIR light sources positioned on the right hemisphere. Energy deposition within various brain tissues and regions was compared between the AD male and female average phantoms. Sources targeting frontal and temporal regions deposited more energy in the brain parenchyma (consists mainly of white and gray matter) of the male phantom compared to the female phantom, whereas sources for trans-ocular, trans-parietal, and trans-occipital paradigms deposited less energy in the male phantom. The total energy deposition proportions were 1.35% for the male phantom and 1.45% for the female phantom. This framework provides insights into the light propagation pathways within these regions during NIR light treatment, offering valuable guidance for developing targeted interventions with optimized illumination parameters such as wavelength, positions, and directions (Video 2).

### 3.4 Simulation Using Patient-Individualized Phantoms Reveals Potential Dose–Response Relationships

We implemented the established framework to perform the multi-source simulation using the patient-individualized phantoms. The parameters of the illumination system are consistent with those described in Sec. 3.3, and the phantoms were derived from T1WI data collected during the pre-treatment (baseline) and post-treatment (week 12 of treatment) visits in the clinical trial.^24^ The therapeutic effect was evaluated using ADAS-Cog, ADL, and mean changes from baseline on the MMSE at baseline, weeks 4, 8, and 12, demonstrating significantly reduced ADL scores at weeks 8 and 12 and improved MMSE scores at week 12 (t-test, p<0.05) [Figs. S5(a) and S5(b) in the Supplementary Material].

**Fig. 5:**
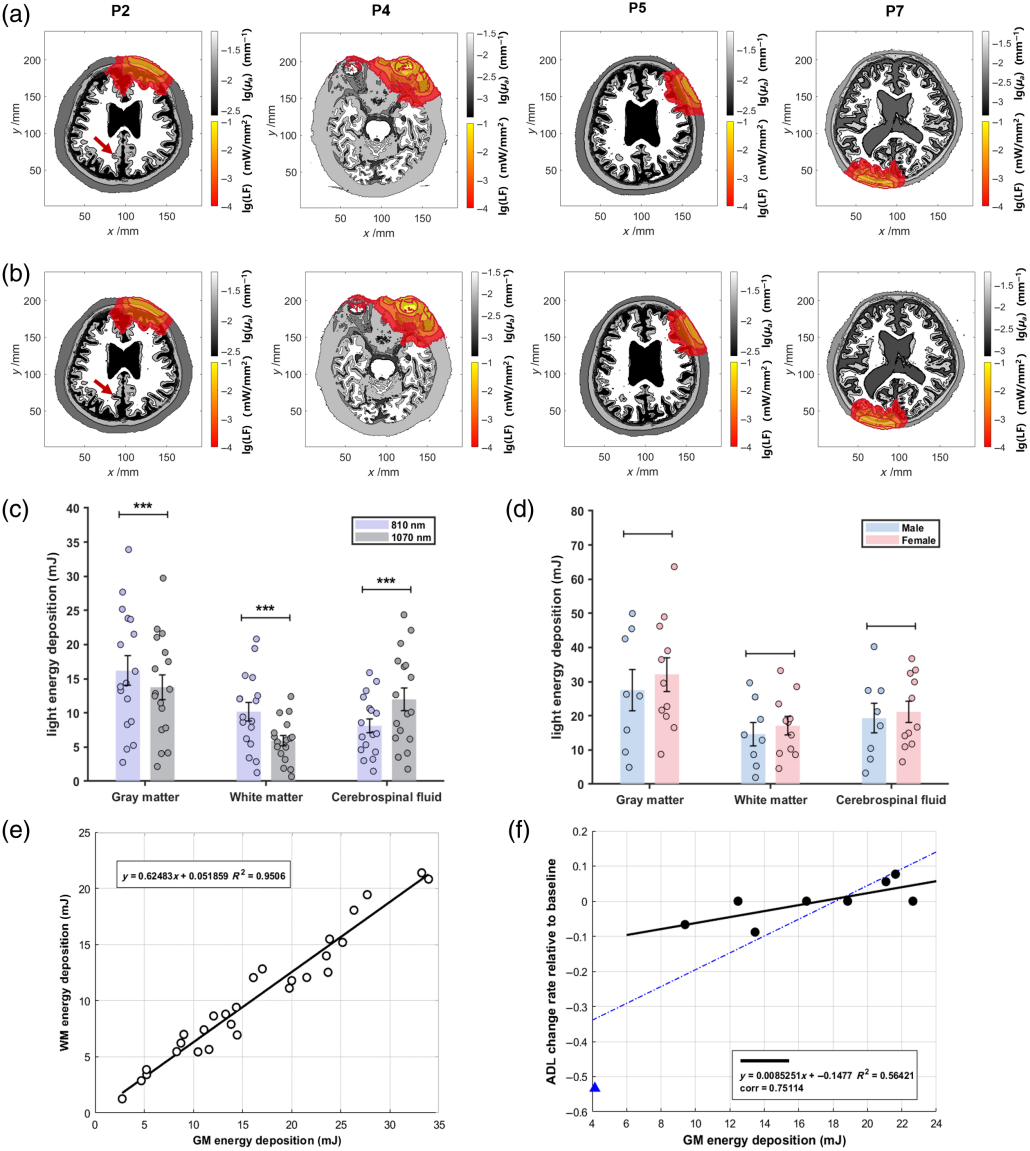
Clinical validation and dose–response analysis using phantoms derived from AD patients who received the NIR light treatment. (a) Horizontal planes of light fluence (LF) distribution and absorption coefficient ($\mu_{a}$) map in the phantoms derived from four AD patients at baseline and (b) at week 12. Red arrow, to highlight dynamic change of optical properties and brain structure of the same patient during the longitudinal study. (c) Energy deposition in gray matter (GM), white matter (WM), and cerebrospinal fluid (CSF) at wavelengths of 810 and 1070 nm (N=17; Kolmogorov–Smirnov test: p>0.05, Levene’s test: p>0.05, two-tailed paired t-test: ***p<0.001; bar height, mean value; error bar, standard error). (d) Sex-based differences in total energy deposition within the brain tissues ($N_{\text{male}}=8$; $N_{\text{female}}=11$; Kolmogorov–Smirnov test: p>0.05, Levene’s test: p>0.05, equal variance t-test: p>0.05; bar height, mean value; error bar, standard error). (e) Linear regression analysis for WM energy deposition over GM energy deposition (N=28; $R^{2}=0.9506$; a (slope) = 0.6248, t-test for a, p(a)<0.001). (f) Linear regression analysis for ADL scale change rate relative to baseline over GM energy deposition at week 8. Dotted blue line: linear fit function using all the samples (N=9, $R^{2}=0.6591$; a (slope) = 0.02398, t-test for a, p(a)=0.0079; correlation coefficient (corr.) = 0.8119; light wavelength, 810 and 1070 nm). The blue triangular data point represents an outlier with an anomalously high baseline ADL score. Solid black line: linear fit function excluding the outlier (N=8, $R^{2}=0.5642$; a (slope) = 0.00852, t-test for a, p(a)=0.0317; corr. = 0.7511; light wavelength, 810 and 1070 nm).

Prior to patient-specific simulations, the phantoms were rigidly registered to the standard MNI space to minimize individual variability and ensure comparability among participants in brain ROI-specific dose quantification. In addition, phantoms derived from week 12 were aligned to their baseline counterparts using the same registration method to enable motion correction and longitudinal analysis [Fig. S5(c) in the Supplementary Material].^41^ This standardized framework preserves anatomical detail, facilitating detailed comparative analysis of light fluence distribution and absorption coefficient maps in phantoms derived from AD patients before and after treatment [Figs. 5(a) and 5(b)]. Subsequently, to identify the interventional dose of the dual-wavelength clinical device, we calculated the wavelength-dependent energy deposition [Fig. 5(c)]. For simulations based on patient phantoms derived from baseline T1WIs, the NIR light sources were normalized to the standard space and configured to deliver identical power densities. The total energy deposition per unit treatment duration in gray matter, white matter, and cerebrospinal fluid revealed that light with a wavelength of 810 nm achieved significantly higher energy deposition in gray and white matter, with less energy loss in cerebrospinal fluid, compared with 1070 nm. This finding was statistically significant (N=17; two-tailed paired t-test: p<0.001). Furthermore, we compared the sex-based differences in total energy deposition within the brain tissues of AD phantoms derived from male and female groups [Fig. 5(d)]. The mean light dose absorbed by the brain tissues in the female AD group was consistently higher than that in the male AD group. The dose data of each group follow a normal distribution (Kolmogorov–Smirnov test: p>0.05) and have equal variances (Levene’s test: p>0.05), but this difference did not reach statistical significance (equal variance t-test: p>0.05) probably due to the limited sample size and individual heterogeneity.

Subsequently, we conducted a linear regression analysis to evaluate the relationship between white matter and gray matter energy deposition within patient phantoms, which indicated a highly collinear relationship during the treatment paradigm ($R^{2}=0.9506$, slope (a)=0.6248) [Fig. 5(e)]. Therefore, we explored the dose–response relationship using linear regression analysis to assess the rate of change in the ADL scale relative to baseline over dual-wavelength (810 and 1070 nm) energy deposition at week 8 [Fig. 5(f)]. Due to the limited sample size of the recorded scale scores at week 8 of the trial, we first used all ADL data collected from the intervention group for linear regression analysis, which demonstrated a fitted slope of 0.02398 with statistical significance (t-test, p(a)=0.0079). However, it is noteworthy that one outlier strongly affected the slope of the fitted primary function. The outlier data were collected from a patient whose baseline ADL score (ADL=30) was significantly higher than the mean score of all participants (ADL=19.0±5.14). Given the potential errors associated with the ADL scale, such as subjective variability in self-report, rater bias, or inaccuracies in the scoring process, we decided to exclude this outlier from the analysis. Linear regression analysis of the remaining data then showed a smaller fitted slope of 0.0085 and also statistical significance (t-test, p(a)=0.0317).

In addition, we identified the structure-related dose distribution induced by illumination sources positioned at different stimuli sites. We used the same clustering methods as in Sec. 3.3 to analyze the energy penetration rate through extracerebral tissues of all the patient phantoms [Fig. S5(d) in the Supplementary Material]. The patients included in the same cluster may have similar intervention outcomes, and it is promising to predict intervention effects based on dose distribution patterns using this simulation framework. For example, patients P5 and P11 have similar trends in ADAS-Cog scores, which were 13, 15, 16, and 16 for P5 and 9, 12, 14, and 14 for P11 at baseline, weeks 4, 8, and 12, respectively. Besides, both the patients had improved MMSE scores at week 12 compared with baseline. In addition, it was shown that light sources symmetrically distributed along the left and right hemispheres of the brain had similar overall interventional energy penetration, and the two light sources targeting the frontal lobe had the highest energy penetration rate through extracerebral tissues at a wavelength of 810 nm. Then, we explored the ROI-specific volume change rate and energy deposition change rate at week 12 relative to baseline in the intervention and control groups [Figs. S5(e) and S5(f) in the Supplementary Material]. The potential associations between AD-related brain ROI volume and NIR light intervention were examined. The intervention group showed a trend for a smaller reduction ratio of average region volume and total energy deposition within the entorhinal cortex compared with the control group. As the entorhinal cortex is one of the first regions to be affected in AD, leading to early symptoms related to memory loss, the results demonstrate the promising therapeutic effect of alleviating the degeneration of the left hemisphere entorhinal cortex. However, because not all patients underwent double MRI examinations at baseline and week 12, these supplementary results were not statistically significant and therefore need to be verified by larger clinical studies in the future.

To further explore the correlational relationship of the therapeutic outcomes across brain region-specific energy deposition and region volume change, we conducted a correlation analysis as shown in the hot map (Fig. S6 in the Supplementary Material). Because only eight patients out of the twenty participants underwent MRI at both baseline and week 12, only eight pairs of phantoms (four from the intervention group and four from the control group) could be used for the ROI-specific dose–response analysis. There is generally a positive correlation between ROI-specific dose and ADAS-Cog or ADL, whereas there is generally a negative correlation between ROI-specific dose and MMSE, which tends to be consistent with the result shown in Fig. 5(f). The Pearson correlation coefficients are statistically significant only in a few brain regions, such as the left hippocampus and inferior temporal for 810 nm and left entorhinal and right amygdala for 1070 nm (two-tailed t-test p<0.05). In addition, the dose–response relationship of the intervention group with wavelengths of 810 and 1070 nm demonstrates similar trends of change in these AD-related brain ROIs. Furthermore, the correlation coefficients of brain ROI volume with therapeutic outcomes in the intervention group indicate that increased ROI volume tends to lead to alleviated ADAS-Cog and ADL scores while contributing to improved MMSE scores. Although the current findings are validated on a limited cohort and require further investigation with larger clinical datasets, the proposed framework demonstrates significant innovative potential and translational promise and provides valuable insights for the advancement of NIR light treatment for AD.

## 4 Discussion

This study focuses on patient-individualized dosimetric simulation and light fluence pathway analysis of NIR light treatment for AD patients. We aimed to enhance pre-treatment precise intervention planning by developing an integrated simulation framework based on the Monte Carlo method and a standard workflow for reconstructing optical-parametric phantoms. By integrating neuroimaging methods, we quantified photonic energy distribution related to illumination parameters, elucidated structure-related correlations of light fluence pathways, and identified potential brain region-specific dose–responses using the simulation results and AD assessment scales.

The preprocessing framework was first validated using extended anatomical templates with comprehensive extracranial tissue structures, enabling simulations for trans-ocular, trans-oral, and trans-nasal treatment paradigms. By combining Hermite interpolation and sparse expert annotations, the framework achieved high anatomical accuracy and fidelity at tissue boundaries, with clear visualization of intricate structures such as the frontal sinuses and visual organs [Figs. 1(e), 2(a), and 2(c)]. Besides, the effective attenuation coefficient ($\mu_{\mathrm{eff}}$) extracted along the illumination direction in the phantom demonstrates layered features of the scalp, skull, cerebrospinal fluid, and gray matter with good continuity and smoothness [Fig. 3(b)]. In practice, the entire synthesis process for a single phantom was completed in 1.5 h, with minimal burden of manual annotations as most steps were automated. To standardize the simulation process, the framework utilized the MNI space, which offers consistent coordinate origins and orientations across individuals, ensuring anatomical comparability and precise light source alignment. A robust registration method was employed to align phantoms to the MNI standard space, facilitating inter-participant and longitudinal analyses. The framework demonstrates scalability and adaptability to diverse T1WI datasets and was validated using AD patient data from our previous clinical study.

NIR light treatment is generally regarded as a mild physical therapy employing low-intensity, non-ionizing, and non-invasive illumination. Although no severe adverse effects have been reported in previous trials,^61^^,^^70^^,^^71^ a comprehensive safety assessment remains critical for its practical application. The simulation in Sec. 3.1 demonstrated that the light fluence rates at the skin surface, cornea, and retina were below the ANSI standards. Although these therapeutic paradigms theoretically show promising efficacy and safety, one mild device-related adverse event was observed in our previous study.^24^ This adverse event is more likely attributable to concomitant diseases, such as diabetes. In addition, the simulation for the trans-ocular paradigm modeled patients’ eyelids as relaxed and naturally closed, as recommended in the clinical study. Consequently, the scattering effect of light through the skin was taken into account. However, there remains the possibility of patients directly gazing at the light source, which could result in light exposure levels exceeding the calculated values. To evaluate the long-term efficacy and safety of this treatment, extensive double-blind clinical studies involving larger cohorts are required. This quantitative simulation framework holds significant potential for assessing and enhancing safety in clinical studies, optimizing energy deposition, and refining treatment protocols to minimize adverse effects while maximizing therapeutic outcomes.

In terms of illumination parameters optimization, we compared multiple treatment paradigms and examined the differences in light fluence distribution across various wavelengths, exploring the potential for high spatiotemporal resolution targeted intervention in specific brain regions. As shown in Fig. 3 and Table 2, we found the transcranial and trans-ocular paradigms more effective for energy delivery to brain tissues. By contrast, the trans-oral and trans-nasal paradigms were less effective, suggesting they are not ideal for direct brain stimulation but may still elicit peripheral effects or remote photobiomodulation (PBM) effects.^72^ Furthermore, our findings reveal that light with wavelengths of 810 to 850 nm achieves the greatest penetration depth, whereas light at 980 nm exhibits the lowest transmittance in the extracerebral tissues of the phantom, primarily influenced by $\mu_{\mathrm{eff}}$, the combined attenuation effect due to light scattering and absorption by turbid tissues. These results correspond to experimental findings from studies conducted separately on mouse models and human extracerebral tissue models.^73^^,^^74^

The simulation results effectively represent subtle spatial variations in tissue anatomy. As illustrated in Fig. 3(f), the non-monotonic behavior of the light fluence rate curve can be attributed to light scattering within biological tissues and boundary effects, including reflection and refraction, which redistribute and concentrate light in certain regions. In addition, the calculated temporal point spread function (tPSF) of the trans-ocular and transcranial pathways allows precise modeling of light pulse propagation and temporal dispersion in brain tissue, offering the potential to optimize time-gated (or temporal interference) treatment for the simultaneous intervention of targeted deep regions using multiple light sources, thereby effectively reducing the accumulation of Aβ plaques and tau tangles in these regions.^75^ This approach holds promise for improving the efficacy and safety of NIR light treatment interventions by minimizing off-target effects and maximizing energy delivery to deep brain regions such as the amygdala and hippocampus. In addition, the Sankey diagrams and correlation heat map [Figs. 3(g), 3(h), and 4(g)] highlight the role of spatial connectivity in light delivery and offer a potential fingerprinting method for predicting therapeutic outcomes based on patient-specific brain networks and dosimetric patterns. The hierarchical clustering revealed distinct clusters of brain regions with similar light propagation pathways and energy distribution patterns. These clusters may represent structurally or functionally connected regions sharing dose–response features.

Furthermore, sex-based differences were evaluated using both average and patient-specific AD phantoms [Figs. 4(h), 4(i), and 5(d)]. Across both types, the total energy deposition proportions in male AD phantoms were marginally lower than those in female AD phantoms. Under identical illumination parameters, structural variations in extracerebral tissues between male and female phantoms were found to significantly influence dose distribution within white matter, gray matter, and specific cortical subregions, including the superior temporal, superior parietal, and lateral occipital regions. These findings underscore the impact of sex-specific anatomical differences on the efficacy and precision of NIR light treatment.

To investigate the therapeutic effects and dose–response relationships of NIR light treatment, this study introduces a novel framework for the precise evaluation of brain ROI-specific dosimetric features and their associations with cognitive and behavioral outcomes, utilizing phantoms annotated with the Desikan–Killiany atlas. This approach provides detailed insights into photon transport pathways and energy deposition within specific brain ROIs across multiple wavelengths. The intervention group receiving NIR light treatment exhibited a significant decline in ADL scores at weeks 8 and 12 and a notable improvement in MMSE scores at week 12 compared with the control group [Figs. S5(a) and S5(b) in the Supplementary Material]. Interestingly, under identical intervention conditions, patients with higher energy deposition in brain gray matter demonstrated limited declines or increases in ADL scores, suggesting a potential interaction between energy deposition and functional outcomes [Fig. 5(f)]. As energy deposition patterns are primarily influenced by patient-specific structural and optical properties of extracerebral and brain tissues, we hypothesize that this phenomenon may stem from neurodegenerative changes caused by AD and structural alterations in the affected brain regions, resulting in suboptimal therapeutic outcomes. To explore the relationship between structural degeneration and dose–response effects, we examined AD-associated volumetric changes and energy accumulation patterns before and after the treatment [Figs. S5(e) and S5(f) in the Supplementary Material]. Although limited statistical significance was observed (likely due to the small cohort), there was a notable trend toward ROI volume increases or mitigation of volume decay, accompanied by volume-dependent decreases in energy accumulation in the intervention group compared with the control group. In addition, the brain ROI-specific correlation analyses for ADAS-cog, ADL, and MMSE scores, as described in Sec. 3.4, partially supported the hypothesis that volume-dependent energy deposition patterns may influence treatment outcomes. Nevertheless, this assessment has several limitations, including incomplete follow up for a small subset of patients, the discrete nature of cognitive scales, and potential measurement errors introduced by manual scoring. These factors reduce its sensitivity to detect subtle functional changes and compromise the robustness of the fitted slope in [Fig. 5(f)]. Therefore, future studies should prioritize large-sample, multicenter, double-blind clinical trials to validate these observations and establish more robust conclusions.

Although the simulation framework demonstrates significant potential to advance NIR light treatment for AD patients, further research is essential to improve its practicality for individualized treatment planning and strengthen its robustness for dose–response analysis. For example, to address the limitations of cognitive scales, neuropsychological assessments could be integrated with multimodal imaging techniques and cerebrospinal fluid analysis, thereby providing complementary functional and biological information. In addition, more extensive basic research is warranted to fully understand the underlying mechanisms and to capture the effects of NIR light treatment on AD patients. Advanced interdisciplinary methodologies could be employed to assess the dose–response relationships of NIR light treatment, including the use of electroencephalogram networks for detailed electrophysiological activity recording and functional near-infrared spectroscopy for hemodynamic monitoring.^76^^–^^78^ Moreover, advanced computational approaches, such as artificial intelligence algorithms, could play an important role in optimizing illumination parameters and refining treatment strategies. The development of personalized, rapid, and adaptive home-based therapies, supported by expert guidance through cloud platforms and remote quality control systems, would enable efficient and responsive treatment planning.^79^ Furthermore, the incorporation of closed-loop modulation modules, such as real-time temperature sensing, neuropsychiatric state extraction, and automated feedback systems, into the dosimetric planning framework could effectively minimize potential thermal effects and improve therapeutic outcomes. As a highly accessible technology, NIR light treatment holds great promise as an adjunctive therapy for AD. Continued innovation of novel therapeutic devices and the establishment of comprehensive treatment strategies are expected to bring tangible benefits to AD patients.

## 5 Conclusion

In summary, we developed a versatile framework for dosimetric evaluation of NIR light treatment and validated it in AD patients, elucidating light fluence pathways and energy deposition patterns in various treatment paradigms. The framework integrates noise reduction in extracerebral tissues, precise cortical segmentation, and sub-cortical parcellation, facilitating detailed mapping of multi-wavelength optical parameters. For the first time, Monte Carlo simulations for time-resolved light propagation were investigated in a comparable-scale cohort of AD patients, moving beyond the reliance on conventional average brain templates. This innovative approach, combined with neuroimaging methods for brain region-specific structural parameter extraction and dose–response relationships analysis, provides insights into the interaction mechanisms of photons with brain structures and opens possibilities for comprehensive investigations such as safety control, parameter optimization, and intervention planning for NIR light treatment.

## Supplementary Material

10.1117/1.NPh.12.1.015010.s01

10.1117/1.NPh.12.1.015010.s1

10.1117/1.NPh.12.1.015010.s2

## Data Availability

All data generated, codes used during this study, and all raw data are available from the corresponding authors upon request.
