# Supplementary material for: Quantitative simulation of near-infrared light treatment for Alzheimer’s disease using patient-individualized optical-parametric phantoms
Source: Neurophotonics. 2025 Feb 18;12(1):015010. doi: 10.1117/1.NPh.12.1.015010 (PMC11833699; doi:10.1117/1.NPh.12.1.015010)
Supplement: Supplementary file 1 [file NPh_012_015010_SD001.pdf]

Supplementary Materials for

•Quantitative Simulation of Near-Infrared Light Treatment for Alzheimer's Disease Using Patient-Individualized Optical-Parametric Phantoms

Sihan Dong et al.

*Corresponding author: Xunbin Wei (xwei@bjmu.edu.cn); Yuanzhen Suo (suoyuanzhen@zju.edu.cn); Jun Xue (20111220084@fudan.edu.cn),

This Supplementary file includes:

Figs. S1 to S6
References (ref. 56 and ref. 80)

Other Supplementary Materials for this manuscript include the following:

Movies S1
Movies S2

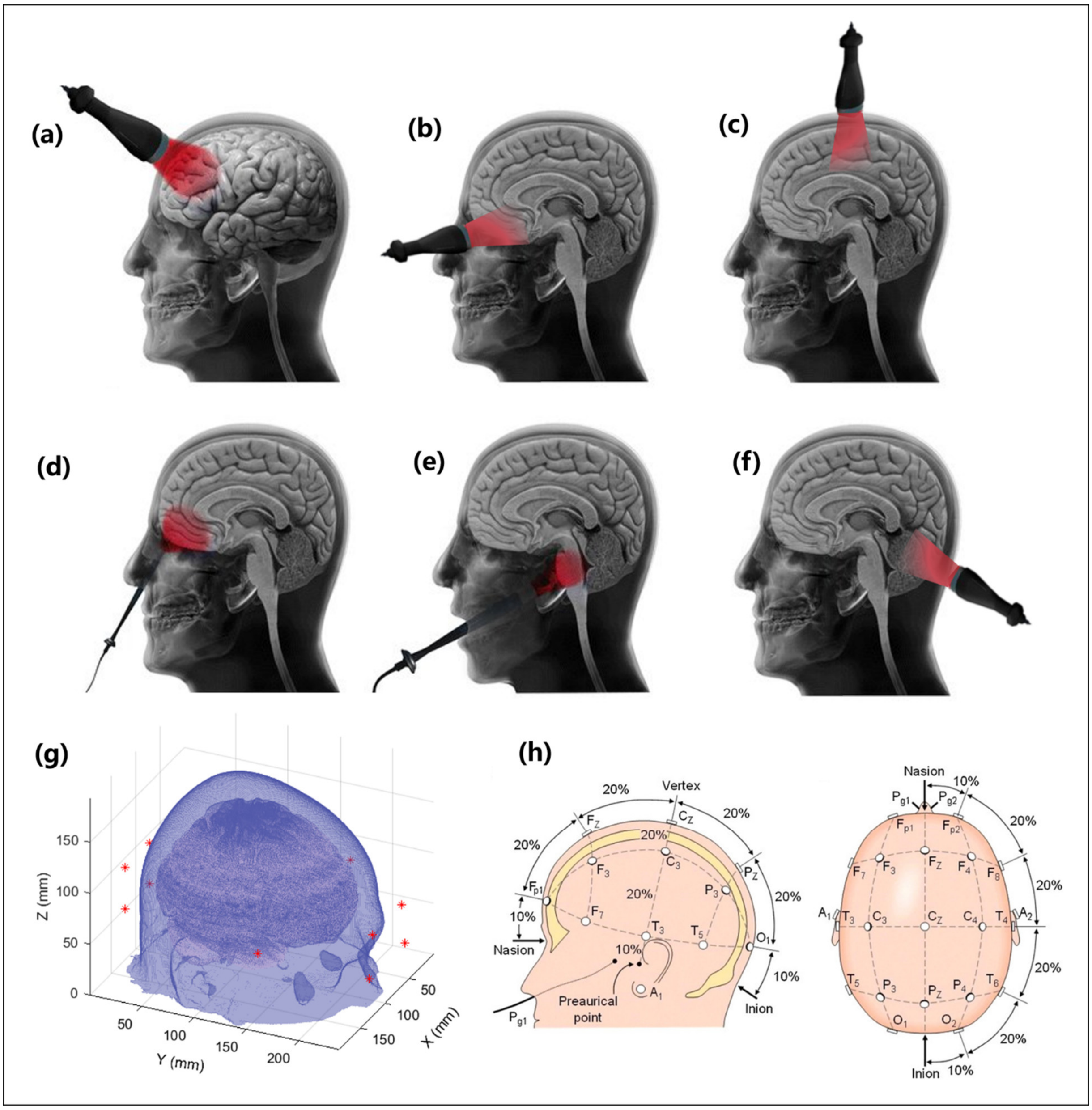

Fig. S1.

Schematic diagram of the simulation setup for the illumination system. (**a-f**) Six typical intervention paradigms: (**a**) transcranial (Forehead), (**b**) trans-ocular, (**c**) transcranial (head top), (**d**) transcranial (occipital), (**e**) trans-oral, and (**f**) trans-nasal paradigm of NIR light treatment. (**g**) The positions of the dual-wavelength light sources used in the clinical study, illustrated by red asterisks attached to the average AD phantom. (**h**) The international 10–20 system for brain stimulation. (a-f) were reproduced with permission from [80] Copyright 2018, Springer Science Business Media, LLC, part of Springer Nature. (h) were reproduced with permission from [56] Copyright 2015, Martins et al.

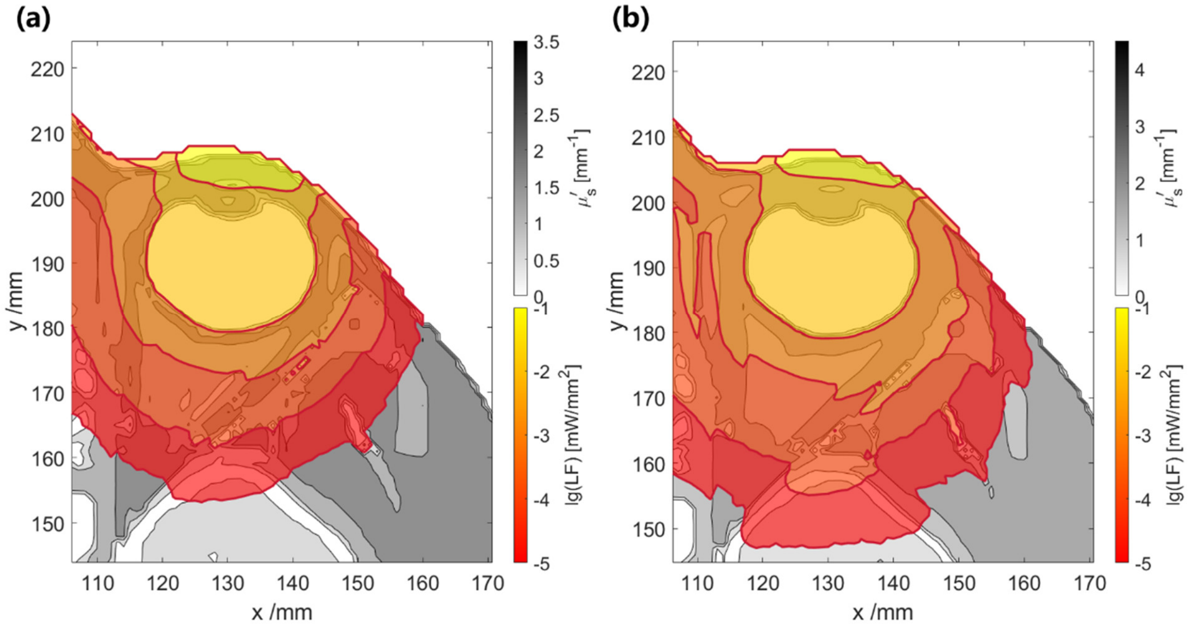

Fig. S2.
Localized magnified contour map of the light fluence rate during simulations of light propagation through the visual organs of the right eye. (**a**) Light fluence (LF) rate at a wavelength of 1070 nm; (**b**) LF rate at a wavelength of 810 nm. (**a-b**) have the same initial power density (20 mW/cm^2) of the light source.

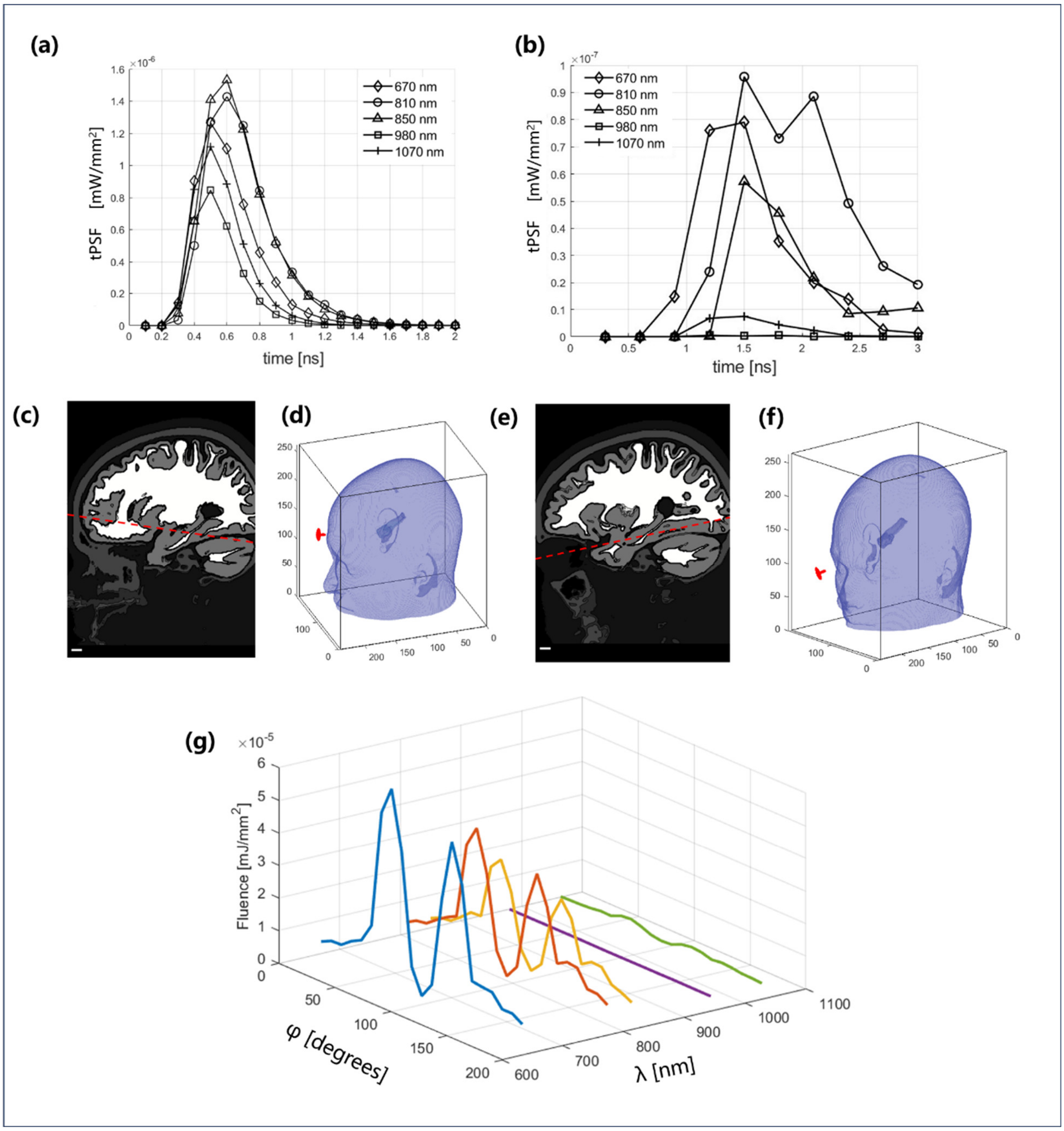

Fig. S3.
Simulation for targeted intervention in deep brain regions under different NIR light treatment paradigms. (**a-b**) Multi-wavelength temporal point spread function (tPSF) in the amygdala region for (**a**) transcranial paradigm and (**b**) trans-ocular paradigm. (**c-f**) Virtual illumination pathways targeting the medial temporal lobe (MTL) region under (c) transcranial paradigm from the forehead and (e) trans-ocular paradigm from the right eye (scale bar: 10 mm); Three-dimensional visualization for relative position of light source and the MTL region in MNI standard space under (d) transcranial paradigm from the forehead and (f) trans-ocular paradigm from the right eye. (**g**) Average light fluence distribution in the brain's hippocampus under different illumination directions (azimuth angle, φ, from 0° to 180°) during trans-ocular treatment.

Right Hemisphere Cortico-Subcortical Network

rh-parsopercularis	rh-precentral	rh-postcentral	rh-transversetemporal
rh-supramarginal	Right-Putamen	rh-insula	rh-middletemporal
rh-superiortemporal	Right-Pallidum	rh-bankssts	Right-vessel
rh-parstriangularis	rh-caudalmiddlefrontal	Right-Inf-Lat-Vent	Right-Amygdala
rh-inferiortemporal	rh-parsorbitalis	rh-temporalpole	Right-Hippocampus
rh-entorhinal	Right-Cerebral-White-Matter	rh-parahippocampal	rh-lateralorbitofrontal
Right-VentralDC	Right-Accumbens-area		

Left Hemisphere Cortico-Subcortical Network

Left-Cerebral-White-Matter	Left-choroid-plexus	Left-Inf-Lat-Vent	Left-Amygdala
Left-vessel	Left-Putamen	lh-insula	lh-middletemporal
lh-superiortemporal	lh-parsopercularis	lh-precentral	lh-transversetemporal
lh-postcentral	Left-Pallidum	lh-parstriangularis	lh-supramarginal
lh-caudalmiddlefrontal	Left-Hippocampus	lh-entorhinal	lh-temporalpole
lh-inferiortemporal	lh-parsorbitalis	Left-VentralDC	lh-bankssts
lh-lateralorbitofrontal	lh-parahippocampal		

Cingulate and Frontal Networks

Left-Lateral-Ventricle	Right-Lateral-Ventricle	Left-Caudate	CC-Central
CC-Mid-Anterior	CC-Anterior	rh-caudalanteriorcingulate	lh-caudalanteriorcingulate
rh-rostralanteriorcingulate	lh-rostralanteriorcingulate	lh-medialorbitofrontal	rh-medialorbitofrontal
CC-Mid-Posterior	lh-posteriorcingulate	rh-posteriorcingulate	rh-rostralmiddlefrontal
rh-superiorfrontal	rh-frontalpole	lh-rostralmiddlefrontal	lh-superiorfrontal
lh-frontalpole	Left-Accumbens-area	Right-Caudate	lh-fusiform
rh-fusiform			

Thalamo-Occipital and Parietal Networks

Left-Thalamus-Proper*	Right-choroid-plexus	4th-Ventricle	lh-lingual
rh-lingual	Right-Thalamus-Proper*	CC-Posterior	rh-pericalcarine
lh-cuneus	lh-precuneus	rh-precuneus	rh-cuneus
lh-isthmuscingulate	rh-isthmuscingulate	rh-inferiorparietal	rh-lateraloccipital
rh-superiorparietal	lh-inferiorparietal	lh-lateraloccipital	lh-superiorparietal
lh-paracentral	rh-paracentral		

Fig. S4.
Desikan-Killiany brain ROIs divided into four main clusters using hierarchical clustering. The corresponding relationship of ROI sorting: from left to right, from top to bottom, corresponds to the ROIs from the top left to the bottom right in the correlation coefficient heatmap of Fig.4 (g).

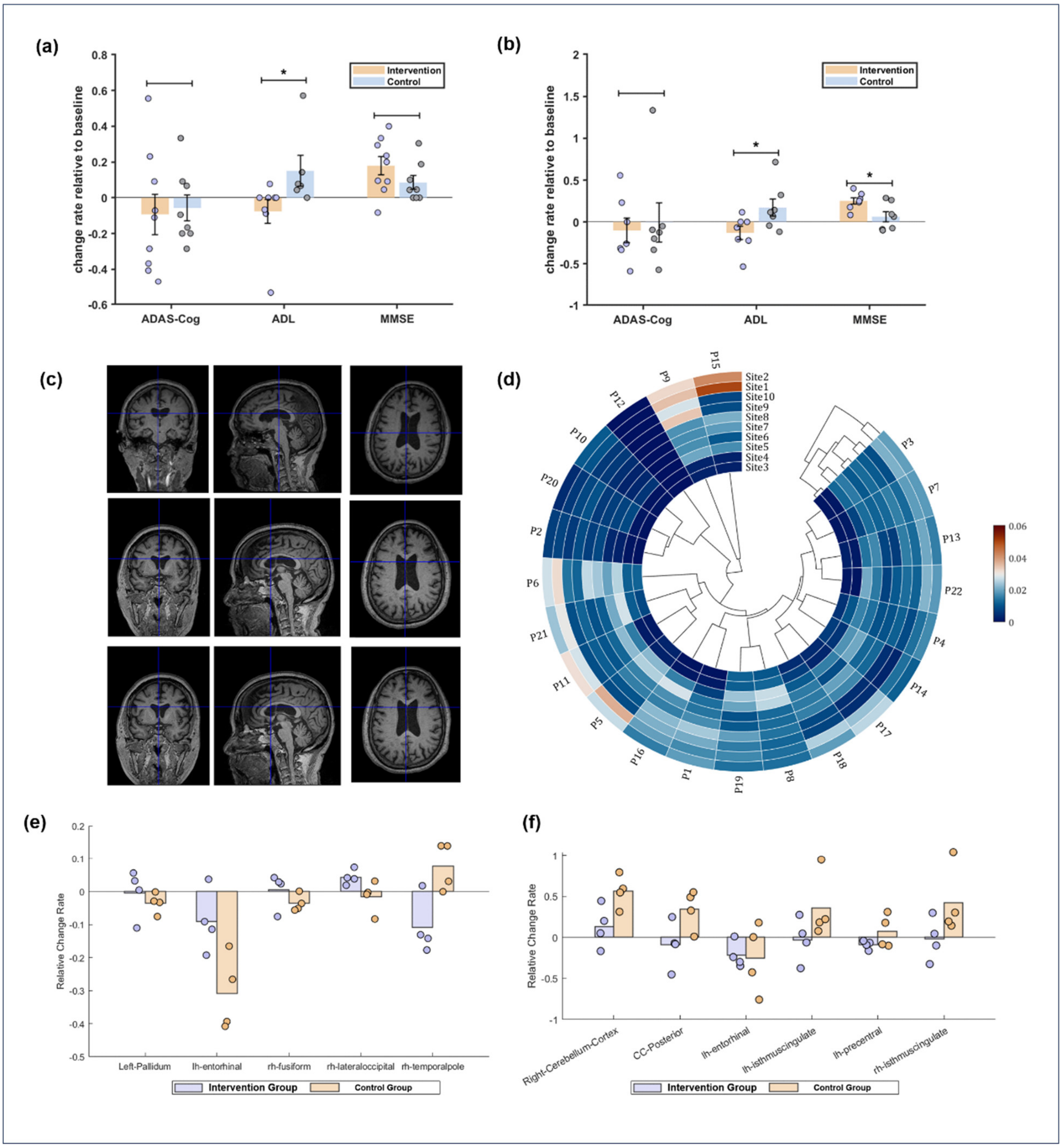

Fig. S5.

Therapeutic efforts evaluated by ADAS-cog, ADL, and MMSE scales (**a**) at week 8 (intervention group, N = 9; control group, N = 8) and (**b**) at week 12 (intervention group, N = 7; control group, N =7); t-test for two independent samples: * $p < 0.05$; bar height, mean value, error bar, standard error (**c**) T1WI data of one participant collected from different visits. From top to bottom: raw T1WI data before registration at week 12; rigidly registered T1WI data after the robust registration at week 12, and the target baseline T1WI data. (**d**) The patient-specific normalized energy penetration rate through extracerebral tissues at a wavelength of 810 nm. P, patients index; Site, irradiation sites: Site number, 1/2: R/L frontal; 3/4: R/L ocular; 5/6: R/L tempus; 7/8: R/L parietal; and 9/10: R/L occipital. (**e-f**) The brain ROIs-specific (e) volume change rate and (f) total energy

deposition change rate relative to baseline in the intervention (N = 4) and control groups (N = 4) with dual-wavelength treatment of 810 nm and 1070 nm. The brain ROIs demonstrating statistically significant pre- to post-treatment changes ($p < 0.05$, ANOVA) in either the intervention or control groups were selected for visualization in this analysis.

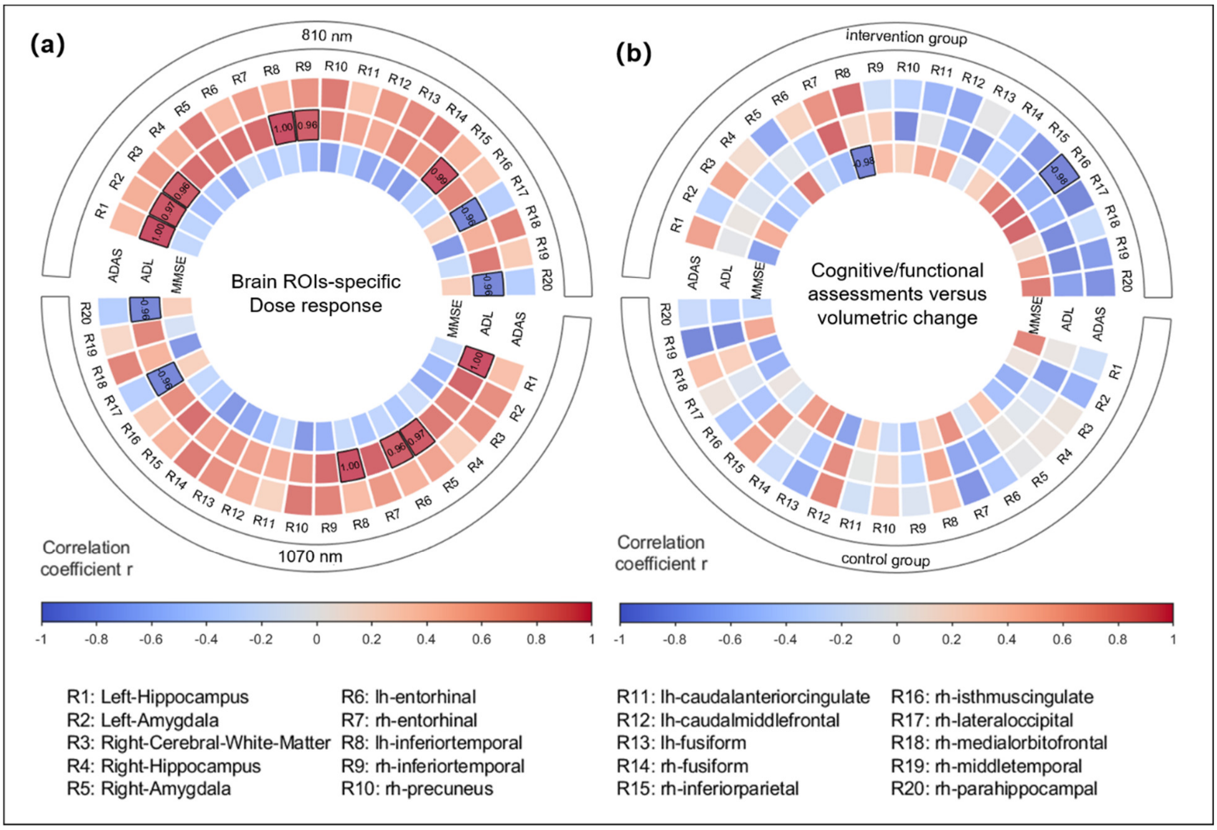

Fig. S6.

Correlation analysis of the brain ROIs-specific energy deposition and region volume with the NIR light treatment outcomes. (**a**) Dose-response relationship of the intervention group with wavelengths of 810 nm and 1070 nm (intervention group, N = 9), calculated using Pearson correlation coefficient. (**b**) Correlation analysis of volumetric change with therapeutic outcomes (intervention group, N = 4; control group, N = 4). Cells with highlighted black border: $p < 0.05$ (two-tailed t-test).

Movie S1.
Tomographic map of horizontal light fluence distribution and absorption coefficient map in the phantoms derived from an average health human template in the standard space (z step: 1 mm, light wavelength: 810 nm).

Movie S2.
The horizontal plane of light fluence distribution and absorption coefficient map for different illumination directions in MNI standard space (φ step: 0.05 π radian, light wavelength: 810 nm).
